# Integrated 3D Mapping and Diagnosis for the Structural Assessment of Architectural Heritage: Morano’s Parabolic Arch

**DOI:** 10.3390/s23146532

**Published:** 2023-07-19

**Authors:** Rosario Ceravolo, Stefano Invernizzi, Erica Lenticchia, Irene Matteini, Giacomo Patrucco, Antonia Spanò

**Affiliations:** 1 Department of Structural, Geotechnical and Building Engineering, Politecnico di Torino, Corso Duca degli Abruzzi 24, 10129 Torino, Italy; rosario.ceravolo@polito.it (R.C.); erica.lenticchia@polito.it (E.L.); 2 Weitzman School of Design, University of Pensylvania, 102 Meyerson Hall, 210 South 34th Street, Philadelphia, PA 19104, USA; matteini@design.upenn.edu; 3 Department of Architecture and Design, Politecnico di Torino, Viale Mattioli 39, 10125 Torino, Italy; giacomo.patrucco@polito.it (G.P.); antonia.spano@polito.it (A.S.); 4 Polito FULL—The Future Urban Legacy Lab, Toolbox Coworking, Politecnico di Torino, 10134 Torino, Italy

**Keywords:** non-destructive techniques, monitoring, concrete retrofitting, 3D mapping, thermal modeling, TIR images, industrial cultural heritage

## Abstract

The architectural heritage of the 20th century is affected by several conservation problems in terms of material preservation, structural analysis, and reuse. Among these, material degradation and durability issues are the ones that have the most effect on the health state and, consequently, the survival of the constructions of the period. In order to conduct a proper analysis for preservation purposes, an interdisciplinary approach is necessary. The parabolic arch in Morano sul Po (Italy) is a reinforced concrete landmark in the Casale Monferrato area and is related to the industrial vocation of the territory, which is indissolubly linked to the cement production chain. The present paper reports the results of a non-destructive test campaign by a Politecnico di Torino multidisciplinary group, which combined acquisitions using different methods. The paper highlights the importance of a structured procedure to integrate different information coming from different techniques. The aim was to assess the health state of the structure and define the best procedures for building an information system based on the as-built modeling strategy, which could serve as the basis to provide conservation guidelines.

## 1. Introduction

The analyses of 20th-century architectural heritage pose new problems in terms of material preservation, structural analysis, and reuse [1,2]. The architectural heritage of the 20th century is affected by several conservation problems. In fact, this heritage is usually threatened by neglect and abandonment, mainly due to the difficult recognition of their historical-documentary value by the non-expert and the public.

Among the different fragilities affecting this heritage, one concerns durability issues. Constructions in reinforced concrete have shown a marked vulnerability to aggressive environmental actions, mainly by carbon dioxide in the atmosphere, chlorides in seawater, and sulfates in water and soil. This fragility usually leads to material degradation and is worsened by a lack of maintenance of exposed concrete, possible errors in the design of the construction details, and errors during the construction stage.

Moreover, possible destructive events, including seismic ones, pose an additional threat, especially because these constructions were built based on design criteria that did not consider, or did limitedly, seismic actions, as confirmed by the lack of technical standards of reference at the time of their construction.

According to ICOMOS standards, conserving modern heritage structures must be based on anamnesis, diagnosis, therapy, and control [3].

It requires a thorough documentation process to gather all kinds of useful information, as well as a multidisciplinary approach to clarify the most controversial aspects. In fact, international guidelines state that the conservation, strengthening, and restoration of architectural heritage requires a multidisciplinary approach [4,5].

A methodological analysis of these structures requires investigations that are directed toward the study of their architecture, geometric morphology and structure, and materials and construction techniques; this analysis is initially supported by historical and archival documents, and then further investigated via direct observation, a 3D survey, and structural analysis. The goal is to highlight the main critical issues of the structure and to provide the best strategies for the conservation and restoration of the buildings in order to reuse them without ruining their original features.

In this framework, structural investigations require highly accurate point clouds for 3D surface reconstruction, but at the same time, a balanced density of information and an adequate level of detail. The 3D model processing, starting from the accuracy validation and the registration of the data derived from different techniques, aims to obtain a unique point cloud model that accurately describes analyzed objects. In other words, according to this objective, the 3D model has to describe a proper general configuration of the building and the precise geometry of the structural elements must be carefully harmonized in order to provide the capability to highlight and analyze anomalies, as well as to ensure data usability and interoperability.

This paper illustrates an integrated approach to 3D mapping and structural diagnosis by means of non-destructive testing for the preservation of a historical industrial monument: the parabolic arch of Morano sul Po. This paper reports the results of the non-destructive test campaign carried out on the structure and focuses on the procedures for structuring an integrated approach, which served as the basis to provide guidelines for its conservation.

### The Arch of Morano sul Po (Italy)

**Figure 1:**
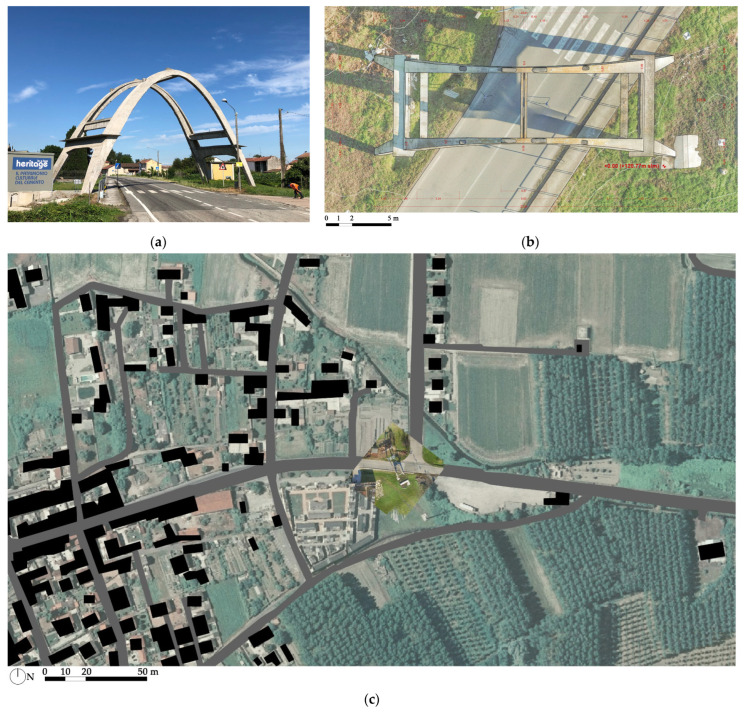
(**a**) Current view of Morano’s Arch; (**b**) plan view from a UAV-photogrammetry-based orthophoto; (**c**) UAV orthophoto (0.005 m) overlaid on a regional orthophoto (GSD: 0.5 m), with buildings (black) and roads (grey) from a regional CTRN.

**Figure 2:**
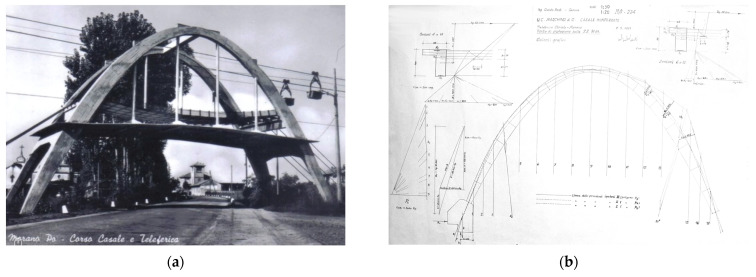
(**a**) Historical postcard showing the original configuration of the arch, where the shielding roofs were present; (**b**) original drawing with the graphic tracing of the thrust line of the arch.

The arch of Morano sul Po (Italy) is located in the suburbs of the city of Casale Moferrato, along the state road no. 31bis (Figure 1). The parabolic concrete arch, together with the protection structure that was dismantled at the end of the last century, was conceived and realized to protect the road from the potential falling of the trolleys, or the carried raw material, of the cableway that is used to connect the mine sites in Coniolo Vecchio and Borino with the factory sites of Morano sul Po [6]. Figure 2a, which was taken from a historical postcard, shows the original configuration of the arch. The design of the concrete arch was appointed to Ing. Guido Sarti, who also provided the structural design of other parts of the cement factory, such as the clinker storage hall and silos, together with the design of the new cableway, which was realized in the period between 1951 and 1952, adopted the same path of the existing one, and was designed by Ing. Gualino in 1908. The structure is similar to one of the first parabolic clinker warehouses, built in the 1920s in the close town of Casale Monferrato, and that had recently undergone an extensive experimental campaign for its condition assessment [7,8].

Fortunately, some of the structural drawings of the arch are still available. Figure 2b shows an excerpt of such design drawings, from which is possible to recognize the graphical procedure for the determination of the thrust line of the arch, which was made with three distinct loading configurations. In addition, the graphical determination of the neutral axis and the stress field in some of the arch sections due to eccentric compression was obtained using the Mohr–Guidi method.

In 2018, following a donation, the structure returned to the ownership of the Municipality of Morano after about forty years. In the summer of 2020, to avert the risk of falling fragments on the roadway, the removal of unstable surface layers was conducted on the arch using a pressure washer. In addition, the exposed reinforcing bars were treated with passivating paint. Recently, thanks to a grant from the Luoghi della Cultura 2018 call of the Compagnia di San Paolo, the structure became part of the Concrete Heritage Box initiative (Figure 1a) [9].

## 2. Multi-Sensor 3D Survey and Mapping for CH Metric Documentation

For several years, there has been a widespread awareness that photogrammetric (image-based) and laser scanning (range-based) technologies, which are often combined, can provide fundamental support to the knowledge processes that invest in cultural heritage, especially architectural heritage, and are therefore fundamental for founding intervention and conservation projects [10].

The versatility of the different sensors, such as aerial, terrestrial, and even those involving acquisition in movement configurations, provides various portable solutions that above all involve SLAM (simultaneous localization and mapping) technology to determine movement trajectories and increasingly lead to being able to document different aspects, such as geometric and radiometric, giving rise to the ever-increasing possibilities of contributing to the knowledge and material characterization of the surfaces and degradation of materials [11,12].

If it is ascertained that many aspects of the reality-based techniques that we now consider traditional are largely established, and since the automation imprinted by the SfM (structure from motion) techniques [13,14] have allowed for the photogrammetric techniques to be able to supply point clouds substantially similar to the LiDAR (light detection and ranging) ones, attention can now be moved to the innovation of processing processes, which often include optimizing hybrid systems and allowing for obtaining multi-sensor and multi-content surface models [15,16].

Co-registration techniques based on ICP (iterative closest point) algorithms often allow for optimizing the results of multi-sensor acquisitions, which may include both multispectral and thermal data, and which can be managed through processes of so-called fusion of techniques, together with visible ones [17,18].

For many of these reasons, the investigation processes that aim to assess the health state of buildings and investigate the material degradation of the construction and structural systems, particularly regarding anti-seismic properties, display a productive operational alliance between the results of the geomatic studies that offer dense and accurate 3D models that are capable of mapping anomalies or supporting the identification of mechanisms of damage or deterioration and are conducted and interpreted through other non-destructive diagnostic investigations by the structural engineering community [19]. It is necessary to find appropriate balances in the information density to simplify processes, and thus, they have to be critically guided in an interdisciplinary discussion [20,21], which is effective for the development of further static or dynamic experiments in a predictive manner and research is providing encouraging results.

Another important opportunity involves the condition of being able to develop multi-temporal investigations, which can document salient moments, both in the life of the artifact and in the restoration or conservation processes [22] that are envisaged and are increasingly linked to the concept of preventive conservation [23]. In this scenario, the unique and stable reference systems over time, in addition to guaranteeing the ability to control the quality of metric data, allow for the future development of the surveys, permitting comparisons spread over time.

The research presented in this contribution exploited integrated reality-based models that relied, as usual, mainly on the laser scanning technique for the geometric characterization of the investigated artifact and its parts, and on digital photogrammetry to investigate the characteristics of the state of conservation of the materials and their degradation. These results served as the basic knowledge to initiate the already mentioned cleaning interventions and the start of the diagnostic assessments on the state of conservation.

Since the artifact being investigated allows for finding good levels of conservation, a thermal survey was also undertaken to clarify how to solve the fusion of geometric and radiometric data in a 3D model that were derived from photogrammetric processes based on images acquired in the visible range of the electromagnetic spectrum and a photogrammetrically controlled texture created from infrared (IR) data.

### Digital 3D Mapping Survey of Morano’s Arch

In 2019, a complete LiDAR survey and a series of photogrammetric acquisitions (both terrestrial and from a UAV) were performed in order to provide multi-sensor three-dimensional documentation of the reinforced concrete asset with the aim of documenting the conservation status of the paraboloid arch in the framework of the restoration works in the planning stage [24].

The laser scanning acquisitions were particularly numerous (19 scans), as in the context of this historic asset with an unusual shape and large openings, the aim was to obtain a high accuracy and metric quality of the surface characterization of the different parts of the arch that had exposed iron in numerous parts of the intrados and extrados, it was decided to experiment with the use of a telescopic pneumatic pole to raise the position of the scans.

The influence of the angle of incidence of the laser beam on the surveyed object, together with the distance between the laser scanner and the object, have been topical issues, with the aim to evaluate how much they affect the quality of the acquired point cloud [25,26], in addition to the influence of the detected materials using range scanners [27]. The adoption of a roughness analysis during the post-processing phase revealed that, unfortunately, the surface of the telescopic pole point cloud was affected by a topological error due to the vibrations transmitted from the laser scanner to the pole during the acquisition of the scan.

**Figure 3:**
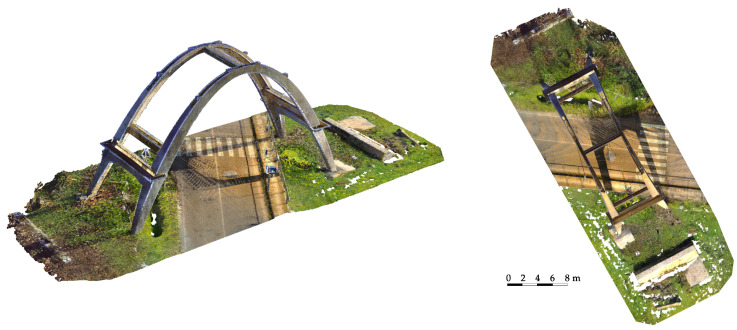
Two views of the LiDAR point cloud derived from the registration of the 19 acquired scans.

**Figure 4:**
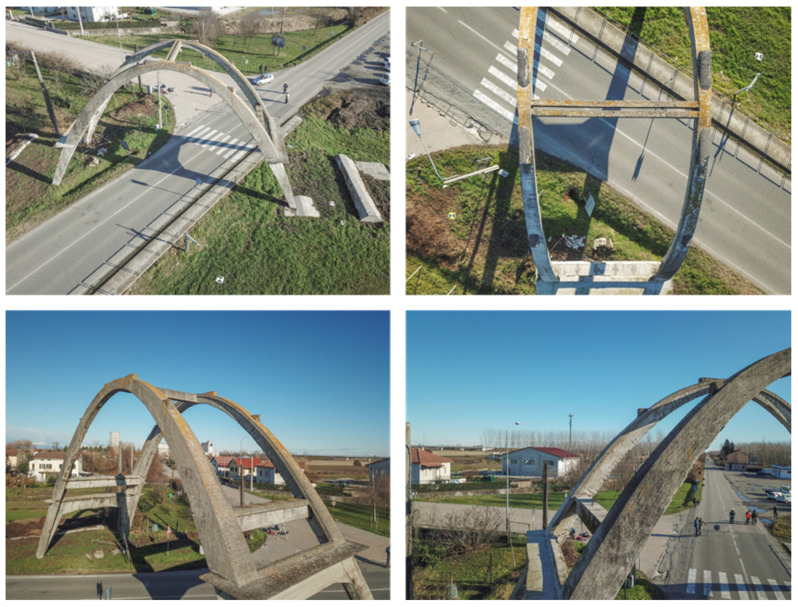
Digital images acquired from an aerial perspective using the micro UAV system, which enabled the acquisition of the extrados and the higher surfaces of the concrete arch.

**Figure 5:**
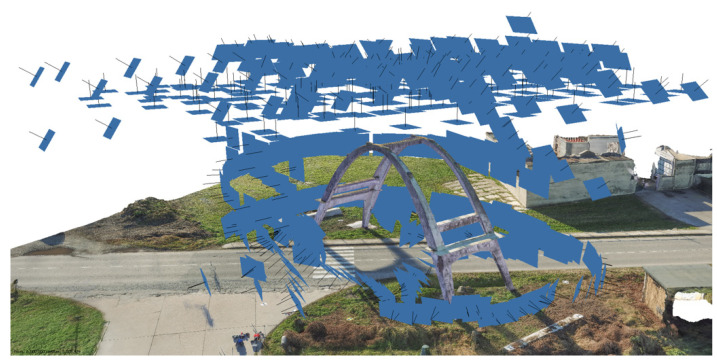
Oriented images (after the relative and absolute orientation) and photogrammetric dense point cloud.

The three main steps of the multisensor survey are briefly described in the following points, which were aligned with a consolidated practice:

1. The possibility of integrating laser scanning and aerial UAV (unmanned aerial vehicle) surveys, as well as subsequently using fusion techniques with thermal data, are linked to fixing a topographic network of vertices, which together with the 3D coordinates determination of control points, is usually aimed at the metric survey of architectural assets to obtain the reference of all measurements and survey documents to a single Cartesian system, as well as to control the propagation of errors in order to guarantee the required tolerances. The vertices were measured with a GNSS (global navigation satellite system) technique in static mode and the calculation was performed using the WGS84-ETRF2000 reference system. From the topographic vertices, a set of control points (both natural points and checkboard markers) were measured using a total station.
2. The LiDAR scans were merged (Figure 3) by exploiting the consolidated registration strategy, which consists of a preliminary alignment of the point clouds using an ICP algorithm, and then a rigid roto-translation of the block composed by the registered scans using the coordinates of the measured markers. The results were close to the usual residuals for similar surveys: the observed accuracy of the control points was ≈6 mm.
3. The photogrammetric survey was carried out by integrating the acquisitions from both a micro UAV (Figure 4) and a DSLR (digital single lens reflex) camera using all of the images (more than 600). Regarding the image-based approach, an SfM solution was adopted. This type of 3D reconstruction is widely used in the field of image-based documentation of heritage buildings since it allows for the generation of dense and accurate point clouds that are characterized by high-resolution radiometry. This feature represents a very useful tool for restorers and conservators since it enables them to consider elements related to the material consistency of a building, thus facilitating its study and diagnostic investigations. The Agisoft Metashape platform (Version 1.8.1) was employed for this operation since it is one of the most commonly adopted and user-oriented software solutions in the field of image-based documentation of heritage buildings. The subsequent pipeline was managed without criticalities (Table 1): image masking, interior orientation of the camera, relative orientation of the images and tie-points extraction, georeferencing and bundle adjustment using the acquired ground control points, accuracy evaluation using both control and check points, and dense point cloud generation (Figure 5).

In particular, in this case, the LiDAR acquisitions provided a point cloud characterized by a very high level of detail and geometric resolution, while the photogrammetric data—derived from the integration of aerial and terrestrial acquisitions—represented the starting point for the subsequent phase of degradation mapping. The high-resolution radiometry that characterized the acquired images and the derived 2D and 3D metric products (orthophotos, textured 3D mesh, etc.) enabled the possibility to efficiently describe features connected to the material consistency or to identify different types of degradation recognizable from radiometric data [15].

In this case, an RGB-based semi-automatic classification was performed with the aim to identify the areas characterized by the following degradations:

- Uncovered irons;
- Efflorescence;
- Pollution blackening.

**Figure 6:**
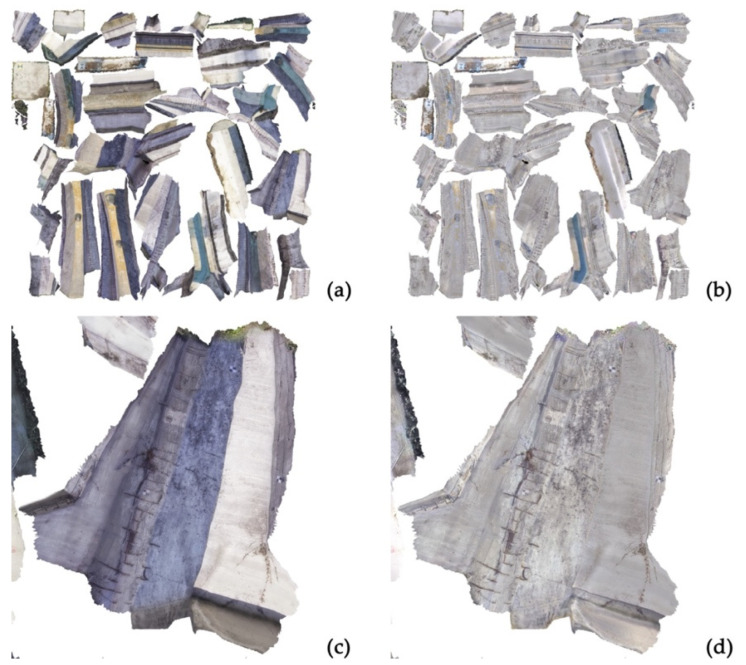
(**a**) Original UV map; (**b**) UV map after the editing process; (**c**) detail of the original UV map; (**d**) detail of the UV map after the editing process.

In order to facilitate the RGB reclassification for the semi-automatic decay detection procedure, the photogrammetric texture was edited with the aim of removing the shadows—due to the different sun exposures characterizing the different surveyed surfaces of the arch—and the radiometric discrepancies caused by the non-homogeneous illumination. As often happens when the data collection is performed in outdoor spaces, the impossibility of managing the lighting may cause the presence of areas characterized by significant differences in terms of radiometry. This can represent a problem for the reclassification task, where the RGB discrepancies are crucial for the proper detection of the analyzed degradation. For this reason, a 3D mesh was computed and a high-resolution UV map was generated (Figure 6). UV mapping is a texturing methodology that consists of projecting a 2D image—namely, the UV map—onto a 3D surface. The UV map is generated by processing oriented images of the photogrammetric block through orthorectifying and mosaicking procedures. Each point on the 2D textured surface corresponds to a vertex on the 3D mesh and is assigned a three-dimensional position, allowing for the texture to be projected onto the model. The UV space uses the Cartesian axes called U and V, while the 3D model is positioned in the XYZ reference system [28].

The generated UV map was processed with the aim of performing a radiometric equalization to homogenize the radiometry of composite elements of the same material but characterized by different solar exposures. This operation was carried out using the Agisoft Delighter platform, which allows for performing the equalization procedure by identifying different samples of underexposed and overexposed areas. The results of this operation can be observed in Figure 6d.

**Figure 7:**
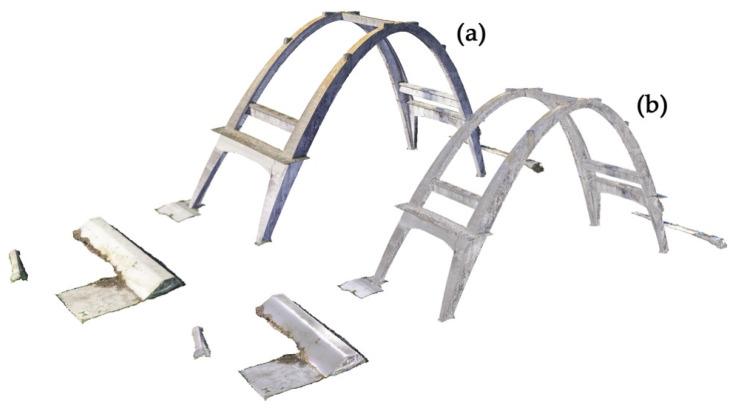
(**a**) Photogrammetric 3D mesh of the arch with the original texture; (**b**) reprojection of the edited texture on the original photogrammetric 3D mesh.

**Figure 8:**
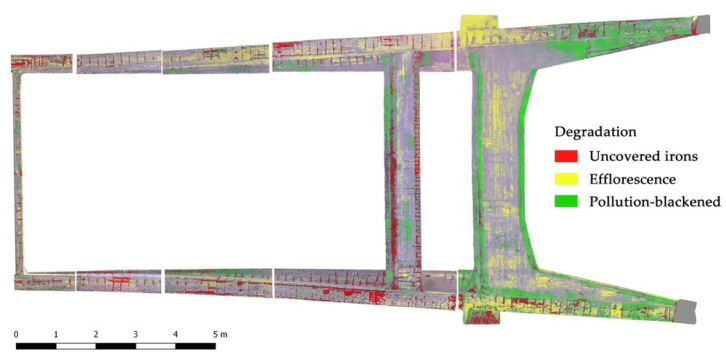
Example of the RGB-based reclassification of a portion of the intrados for the semi-automatic identification of different kinds of degradation.

The edited texture was re-projected onto the computed 3D mesh (Figure 7) for the subsequent generation of the orthophotos that were used as primary data for the RGB-based reclassification for the identification of the analyzed types of decay. The reclassification of the exported raster images was carried out using the open-source software QGIS, which allowed—through the Raster Calculator tool—for grouping clusters of pixels with comparable digital number values and reclassifying them, exploiting the difference in terms of radiometry of the analyzed material (concrete) and decays (uncovered irons, efflorescence, and pollution blackening). After the reclassification processing, a degradation map was generated. In this case, the digital numbers embedded in each pixel of the classified orthophotos contained only four values: 0 for the concrete, 1 for the exposed irons, 2 for the areas affected by efflorescence, and 3 for the areas affected by the presence of pollution blackening (Figure 8).

## 3. Diagnostic Investigation

With the aim to finalize an exhaustive characterization of the materials and structures, the estimation of the type and number of necessary tests was defined on the basis of preliminary assessments and in relation to the construction phases of the work, the static role of the various structural elements, their function with regard to safety the structure, and the degree of homogeneity of the results of any preliminary tests.

In order to characterize the mechanical properties and the health state of the structure, the following tests were carried out:

- Sclerometric tests (n = 23);
- Homeosurface ultrasonic tests (n = 13);
- Pacometric tests on each structural element;
- In situ carbonation tests (n = 4);
- Mapping of the corrosion potential in selected areas (n = 4);
- Monitoring of the internal temperature and humidity of a selected element (n = 1).

The tests were extended for the main structural elements in order to have a uniformly distributed and representative overview of the whole structure. It is important to highlight that only non-destructive testing was employed. The results of these investigations are reported in the following paragraphs and informed the 3D mechanical model built on the basis of the geometrical surveys.

### 3.1. Structural Survey

**Figure 9:**
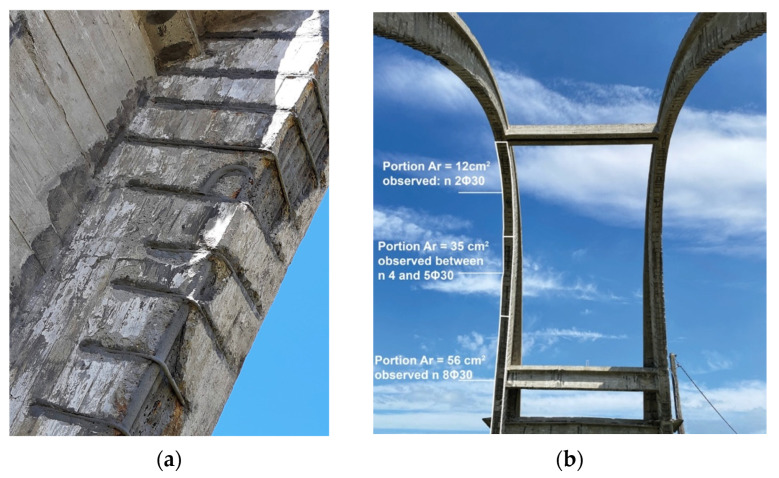
(**a**) Detail of the reinforcements of the arch; (**b**) measurements of surveyed reinforcements in the different portions of the arches.

Pacometric tests allow for non-destructive verification of the presence and number of reinforcements in the structural elements of the work, as well as their orientation and arrangement, while also being able to estimate the thickness of the cover of the investigated elements. It was possible to directly measure the diameter of some of the outermost bars since the iron cover layer was absent in some places of the structure. Further comparison in terms of overall reinforcement area was possible from the design drawings (Figure 2b). The reinforcing bars were smooth with a diameter of 30 mm and had overlapping areas with hooked ends (Figure 9a). The stirrups, also smooth, had a pitch equal to 25 cm and a diameter of 12 mm. Pacometry made it possible to verify the presence of rebar even in areas where the cover was intact.

Consistent with the design reported in the original drawings, it was observed that the percentage of longitudinal reinforcement decreased from the base toward the key of the arch (Figure 9b).

### 3.2. Ultrasonic Pulse Velocity and Rebound Hammer Tests

**Figure 10:**
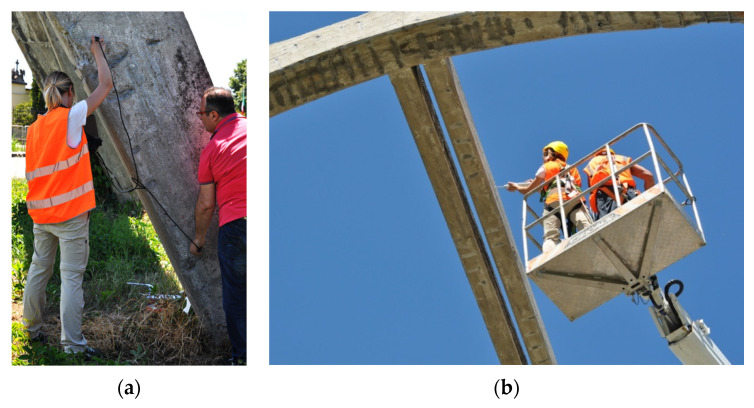
On-site testing of the various elements of the structure: (**a**) ultrasonic pulse velocity test; (**b**) rebound hammer test on one of the top beams connecting the arches.

Ultrasonic pulse velocity tests are well-known non-destructive tests that are used to evaluate the quality and homogeneity of concrete. Measurements were conducted in accordance with the standards [7,10]. The measurement of the propagation time of the ultrasonic pulses was carried out using two probes, namely, a transmitter and a receiver, each with a frequency of 54 kHz, and applied to the surface of the element involved in the test. Indirect, i.e., homeosurface, tests were performed by placing the probes on the same face of the structural element at varying distances (Figure 10a), and once the distance between the points under test was provided, the instrument returned the time and speed of the wave propagation.

The pulse velocity tests were carried out in different representative locations of the structure in order to evaluate possible differences in the cast concrete. The measured pulse velocities are reported in Table 2.

The ultrasonic pulse velocity measurements were then coupled with the results of the rebound hammer tests, which provided a rapid indication of the quality of the concrete.

Although the test is not primarily meant to assess the compressive strength of concrete, when performed in combination with corings, it can be used to estimate the compressive strength through a feasible correlation. However, in the absence of core testing calibration, a lower bound of the compressive strength may be obtained using correlations from the literature.

Measurements were conducted in accordance with the methods proposed in UNI EN 12504-4:2021 [29]. The rebound index was measured by referring to the grids used for ultrasonic tests and positioning the instrument at the center point of each box (Figure 10b). According to the standards, twelve readings for each position were taken; for each set, the lowest and highest values were discarded, and then the average of the ten remaining values was taken. The rebound index I values, which were calculated from the rebound hammer tests, are reported in Table 2.

**Table 2:** Results of the ultrasonic, rebound, and SONREB methods regarding the main elements of the analyzed structure.

Element Number	Structural Element	Pulse Velocity [m/s]	Rebound Index I	Estimated *R*_c_ Method [30] [N/mm^2^]	Estimated *R*_c_ Method [31] [N/mm^2^]	Estimated *R*_c_ Method [32] [N/mm^2^]
1	Base 1 arch	3611	27.1	16.71	18.77	19.83
2	Base 2 arch	3947 4029	31.7 32.8	26.23 29.03	26.90 29.16	29.09 31.72
3	Base 3 arch	4471 3945	28.7 30.02	31.56 24.48	29.93 25.30	35.52 27.60
4	Base 4 arch	3632 3057	41.2 40.1	30.5 18.8	32.0 22.5	31.3 20.0
5	TR 1	3771	36.8	28.71	29.77	30.47
6	TR 2	3992	32.2	27.62	28.01	30.41
7	TR 3	4317 4639	31.9 27.0	33.41 31.89	32.00 29.70	36.46 36.45
8	Arch east	3279	31.6	16.13	19.01	18.42
9	Arch west	4402	30.2	32.55	30.99	36.09

### 3.3. Sonic Rebound

The ultrasonic pulse velocity measurements were then coupled with the results of the rebound hammer tests, which provided a rapid indication of the quality of the concrete. The SONREB (combination of the words sonic and rebound) method is a non-destructive investigation method for use on hardened concrete, which enables the *R*_c_ strength of concrete to be estimated in situ by correlating it with the pulse velocity obtained from ultrasonic tests. More specifically, the velocity obtained from tests using the indirect method, together with the rebound index I obtained from the rebound hammer tests, was used.

It is possible to correlate the strength of the material with the rebound index I obtained from the rebound test and the propagation velocity *V* of the ultrasonic waves by means of experimental mathematical formulas. In fact, there are several experimental formulas in the literature for applying the SONREB method, where the velocity *V* is expressed in m/s and *R*_c_ is the cubic strength of the concrete. However, the experimental formulas all refer to the following general equation:

*R*_c_ = *aI^b^V^c^*

where *a*, *b*, and *c* are the coefficients that allow for the best fit with the direct experimental data. To determine the *R*_c_ strength of the concrete elements of the artifact using the SONREB method, the following formulations were used (Table 3), and the results of the method are given in Table 2.

**Figure 11:**
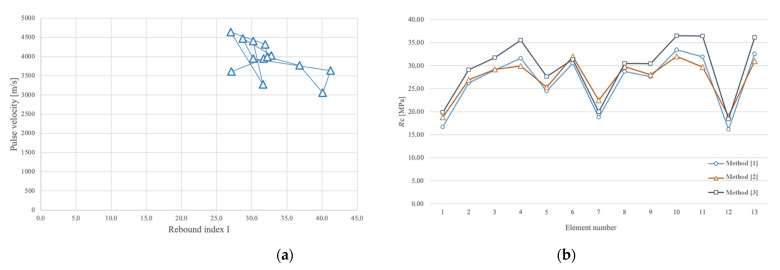
(**a**) Diagram illustrating the values of the pulse velocities and rebound index; (**b**) SONREB results in terms of *R*_c_.

Figure 11a,b shows the results of the SONREB tests in graphical form. As can be seen, the values formed a rather definite cluster, which is generally an indication of a uniform material type.

Moreover, considering the fact that the building was built in the 1950s, it was possible to estimate the mechanical characteristics based on a study of the mechanical characteristics of the concrete of the period. In fact, the work of [33] provides a database reporting the aggregate analyses of the compressive strength values of specimens certificated by the laboratory of material testing of the Politecnico of Torino between 1915 and the early 2000s. By examining four intervals (from 1915 to 1935, from 1936 to 1955, from 1956 to 1975, and from 1976 to 2002), the authors of [33] reported the probability density curves obtained for each of these periods. Based on this study, it was possible to use the expected characteristic resistance of the concrete of the time as a benchmark for comparing it with the results obtained from the SONREB. The expected *R*_c_ of the period was around 20.82 MPa (μ value of both the real and Gaussian distributions). Considering the results of the SONREB methods, it was possible to notice compatibility with the expected *R*_c_ of the time: the results of the NDT testing presented an average value of *R*_c_ that ranged between 26.74 MPa (with method [30]) and 29.49 MPa (with method [32]).

Moreover, based on the results obtained using the SONREB method, it was possible to assert the general uniformity of the concrete characteristics of the various elements of the analyzed Morano’s Arch.

### 3.4. Carbonation Testing

The determination of the depth of carbonation was carried out in accordance with the provisions of EN 14630:2007 [34] using a 1% phenolphthalein solution in ethyl alcohol as a reagent. In order to minimize structural disturbance, the in situ test was conducted by taking advantage of the holes drilled with the percussion drill, which had already been used for direct verification of the cover thickness and for corrosion testing to minimize the removal of the original concrete. Carbonation testing was performed at multiple locations on the columns. At all locations, the carbonation depth was detected to be within 3 mm, as shown in. Given the age of the structure, the limited carbonation depth could be attributed to previous coatings that were installed in the past, as confirmed by the historical photograph (Figure 2a).

### 3.5. Corrosion Testing

A half-cell potential survey was carried out in accordance with the ASTM C876-22b Standard Test Method for Corrosion Potentials of Uncoated Reinforcing Steel in Concrete [35]. The test was conducted by using a copper–copper sulfate reference electrode. Continuity testing was carried out before completing the corrosion testing to assess the presence of electrical continuity within the existing steel reinforcement. Due to limited continuity found between the existing steel reinforcement, testing was carried out in limited areas.

Half-cell potential measurements are expressed in volts or millivolts and provide an indication of the possibility of the corrosion activity present at the time of measurement.

All data collected was evaluated in accordance with ASTM C876 following the guidelines given:

- If potentials over an area are more positive than −200 V (w.r.t. Cu/CuSO_4_), there is a greater than 90% probability that no reinforcing steel corrosion is occurring at the time of measurement of the specific location.
- If potentials over an area are in the range of −200 to −350 V (w.r.t. Cu/CuSO_4_), the presence of corrosion activity of the reinforcing steel is uncertain at the time of measurement of the specific location.
- If potentials over an area are more negative than −350 V (w.r.t. Cu/CuSO_4_), there is a greater than 90% probability that reinforcing steel corrosion is occurring at the time of measurement of the specific location.

**Figure 12:**
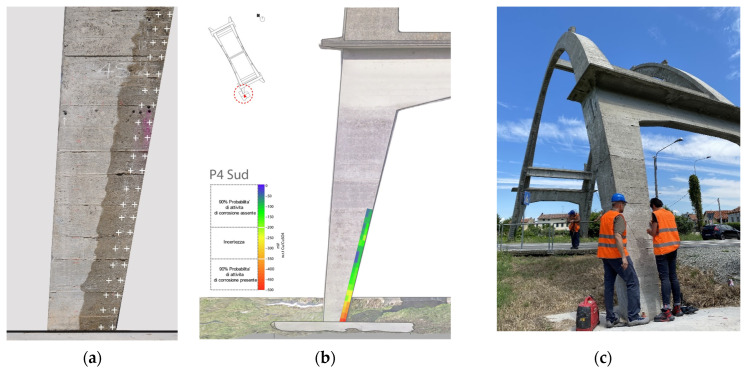
(**a**) Diagram illustrating the grid used to record the potential measurements (“+” indicate measuring locations); (**b**) an example of a half-cell potential map (the red circle specifies the arch pillar); (**c**) a photograph of the team during the fieldwork.

The data collected were translated into color contour maps to better illustrate the findings (Figure 12). Negligible corrosion levels were detected at the time of testing.

### 3.6. Environmental Monitoring

**Figure 13:**
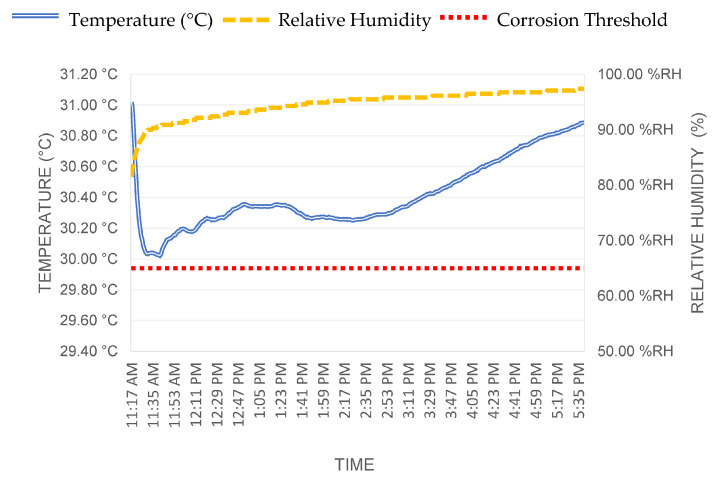
Graph illustrating the data collected of the internal temperature and humidity at the concrete column.

Environmental monitoring is an important factor to consider when assessing reinforced concrete structures. Several of the common deterioration mechanisms that affect reinforced concrete structures are initiated by a certain level of moisture present within the material. With corrosion, moisture is one of the two ingredients, together with oxygen, that needs to be present for the reaction to occur. Internal environmental monitoring was carried out at one location. A temporary sensor was installed inside the concrete to measure the internal temperature and humidity levels. Measurements were recorded every 20 min during the field workday (approximately 5 h). From the internal environmental monitoring, a high moisture content was detected from the data collected on site (Figure 13).

### 3.7. Digital 3D Thermal Survey

Additionally, a complete thermal survey was carried out following photogrammetric criteria with the aim of orienting the TIR (thermal infrared) images in the same reference system of the LiDAR and visible data in order to follow a data fusion strategy that exploited the high geometric resolution of the previously acquired metric data and provide spatial referencing for the thermal information.

The thermal images were collected using a DJI Zenmuse XT2 thermal camera that was equipped on a UAV platform (model: DJI Matrice 200 V2) in order to acquire the higher areas and surfaces of the concrete arch. The employed camera was characterized by the presence of a high-resolution visible sensor, which allowed for the simultaneous acquisition of TIR images and visible images from an approximately constant relative position and an equal angle. The principal characteristics of the two employed sensors are reported in Table 4.

The data were collected while ensuring high convergence of the cameras and a high amount of overlapping (>80–90%) between adjacent images in order to facilitate the extraction of homologous points and, therefore, the relative orientation of the images.

Several UAV flights were performed and a total of 568 thermal images were acquired from an average acquisition distance of 20 m with an estimated GSD (ground sample distance) of 0.36 m. At the same time, a visible dataset—composed of the same number of images—was collected, with an estimated GSD of 0.005 m. Both datasets were subsequently processed using the SfM-based software Agisoft Metashape.

As is well known, there are numerous issues connected to the intrinsic nature of TIR images. These features can significantly affect the use of this kind of imagery for photogrammetric applications, e.g., low spatial resolution, which can cause an insufficient level of detail for an adequate 3D reconstruction; blurred and smoothed-out features where a similar emissivity of adjacent materials is observed, leading to a similar temperature measurement; and difficulties in unambiguously identifying the control points.

**Figure 14:**
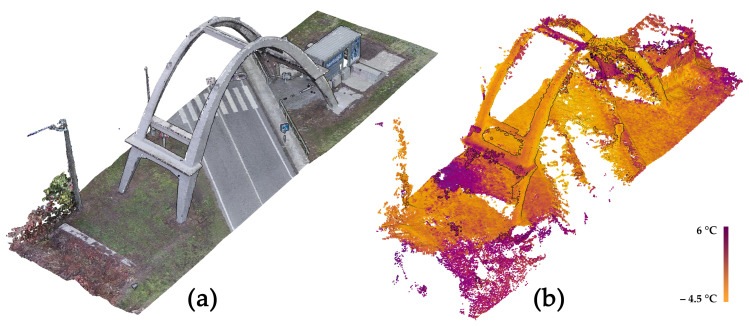
(**a**) Photogrammetric dense point cloud derived from the images acquired from the DJI Zenmuse XT2 visible sensor; (**b**) photogrammetric dense point cloud derived from the TIR images acquired from the DJI Zenmuse XT2 thermal sensor.

For these reasons, in many cases, the 3D reconstruction derived from the photogrammetric use of TIR images was characterized by a high level of noise and by the presence of numerous topological errors, gaps, or lack of information (Figure 14b). Often it is necessary to follow tailored strategies in order to optimize the results derived from the photogrammetric processing of the acquired thermal images. In most cases, a data fusion strategy is highly recommendable since it exploits the high spatial resolution of visible data (Figure 14a) in order to provide spatial referencing for the thermal information.

In the presented case, the following strategy was adopted:

- Interior orientation of the dataset was acquired with the visible sensor of the DJI Zenmuse XT2 camera;
- Absolute orientation was found using a set of GCPs;
- Metric accuracy evaluation was achieved using a set of CPs;
- Importing the relevant camera positions and attitude of the visible dataset images in the project where the TIR images were processed;
- TIR image orientation (using exterior orientation parameters of the processed visible images as an approximate exterior orientation solution);
- Optimization of the orientation using a set of measured GCPs;
- Metric accuracy evaluation on a set of CPs;
- Data fusion between the previously acquired data (LiDAR scans or photogrammetric point clouds derived from visible images) and the TIR data to generate topologically correct added-value metric products (e.g., orthophotos, thermal textured 3D model, etc.).

The set of points used as GCPs and CPs was measured using a total station. In the case of the thermal dataset, both low-emissivity markers—made of aluminum—and easily detectable natural points were measured. Additionally, some additional control points were manually extracted from the LiDAR point cloud.

### 3.8. Data Fusion: Visible and Thermal 3D Modeling

While following the described strategy, it was possible to properly orient the entire visible dataset (568/568 images), and due to the aforementioned issues characterizing the thermal dataset, it was possible to properly orient only 179 TIR images belonging to the thermal dataset. The accuracies observed for the set of measured points used as GCPs and CPs are reported in Table 5.

**Figure 15:**
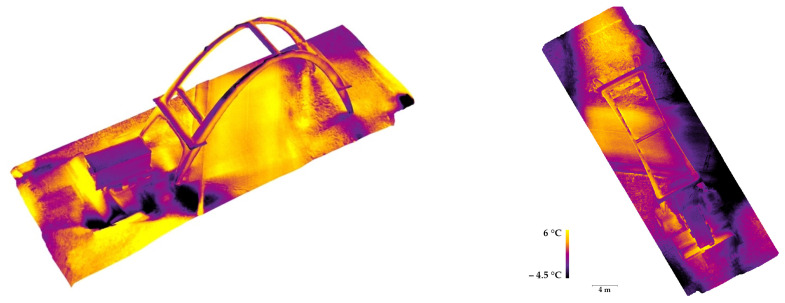
A 3D mesh derived from the integration between the visible data and the oriented TIR images.

Although it was not possible to orient the entire block of thermal images, the described procedure allowed for the generation of high-resolution thermal added-value metric products that were derived via the fusion of the oriented thermograms and the surface model computed from the visible images. Specifically, the following 2D and 3D metric products were generated:

- A high-resolution thermal-textured 3D mesh (Figure 15);
- Thermal orthoimagery (Figure 15).

## 4. Building an Informed Model for the Structural Analyses

Based on the information coming from both the point cloud geometrical survey and the experimental campaign, a 3D finite-element model was built to analyze the structural behavior of the object under analysis. The model was partitioned into macro-components (i.e., mesh subsets) with assumed uniform material characteristics. Each macro-component was finally assembled in the FE software to obtain the mechanical FE model.

The linear elastic FE model of the structure was constituted by mono-, bi-, and tri-dimensional elements: a two-node beam element with 6 degrees of freedom (DoF) in each node, which was described using Timoshenko theory, was used for the reinforced concrete beams at the top of the arch; an eight-node 48-DoF thick shell element with bilinear shape functions was used for the slabs; and an eight-node solid element with 24 DoFs for modeling the arches [36]. The structure was assumed to be clamped at the base of the buttresses, and thus, we neglected the deformability of the foundation.

The FE model was sequentially corroborated using the information that came from the experimental tests to interpret the current structural condition. The mean experimental Young’s moduli were adopted for the macro-elements for which both rebound and ultrasonic tests were available.

To incorporate consistent values in the numerical analyses, the tangent dynamic moduli were estimated via the ultrasonic tests and were used in the FE model, while the static modulus was estimated by using the results of the rebound tests.

Regarding the density, since no data were available on the reinforced concrete specimens, the density of these macro-components was supposed in accordance with National and European regulations equal to 2500 kg/m^3^. Poisson’s ratio was reasonably assumed to be 0.20 for every macro-component. Table 6 reports the values of the mechanical parameters obtained from the experimental tests.

As shown in the previous section, to build the finite element model, all data were used to replicate the mechanical response of the materials. However, it is important to highlight that NDT data may carry a greater amount of uncertainty [37,38] compared with other testing methods. As a result, it was expected that the model also reproduced the static response of the building. However, even if the values of displacements and stresses were detailed, the primary objective of this analysis was to understand the behavior and interaction of the different components of the building under their own load and to compare them.

**Figure 16:**
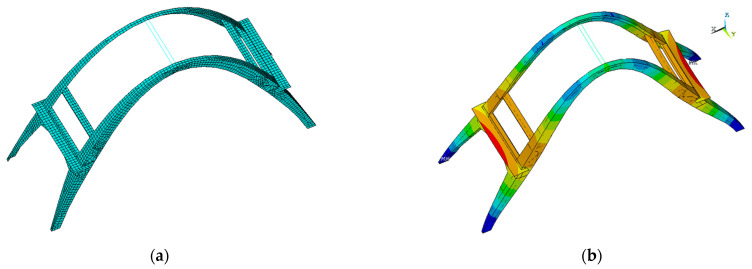
(**a**) View of the 3D finite element modeling of the arch; (**b**) results of the static analysis that identified the static deformations of the structure (the scale goes to blue, deformations close to zero, to red, were the maximum deformations appear).

Figure 16a,b reports a view of the FE meshed model and the results of the static analysis, respectively. Overall, the distribution of deformations and stresses confirmed the substantial symmetry of the structure. The deformed shape of the structure indicated that the arch experienced its maximum deformation around the middle portion of the arches, in particular, at the joint with the beams.

**Figure 17:**
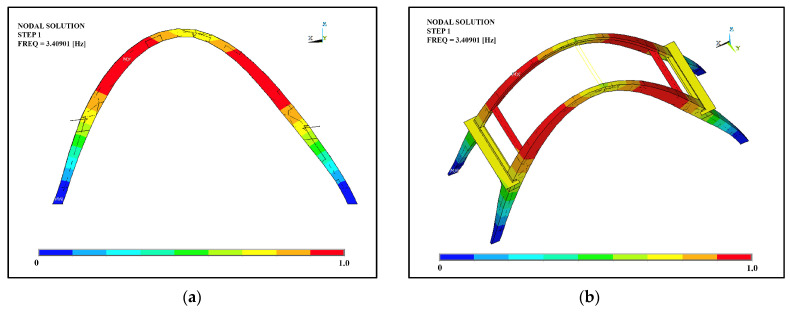
Mode 1: (**a**) lateral view; (**b**) perspective view.

**Figure 18:**
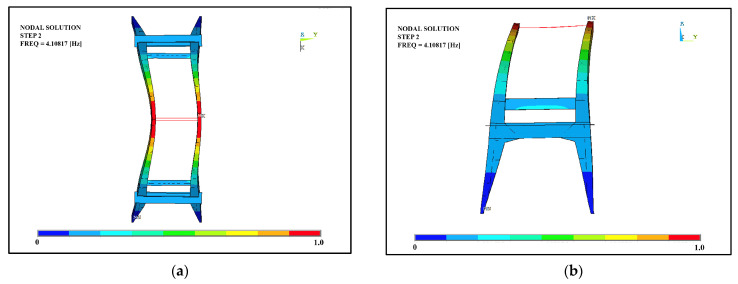
Mode 2: (**a**) top view; (**b**) lateral view.

In order to spot possible criticalities to be better analyzed, structural and eigenvalues analyses were also carried out. Based on an average of all six degrees of freedom, modes 1, 2, 4, 5, and 6 were the modes that excited the most mass; the mode that affected the largest percentage of mass was the first mode, which corresponded with the translational modal form along the x and y directions, with model 2 in second place (Figure 17 and Figure 18). Overall, it can be said that the behavior was governed by the interaction of the arches, which were modelled as being perfectly connected to each other by the presence of the beams. Further experimental tests could investigate the actual connection of these elements to the arches since they could affect both the global and local responses of the structure from a seismic point of view.

## 5. Results and Discussion

Regarding the monitoring results, the arch structure appeared in good condition. Negligible corrosion levels were detected at the time of testing with minimal carbonation depths. Given the age of the structure, the limited carbonation depth could be attributed to previous coatings that were installed in the past, as confirmed by the historical photograph (Figure 2a). From the internal environmental monitoring, high moisture content was detected from the data collected on site. At the top portion of the arch, visible areas of concrete deterioration were observed, showing exposed rebars.

Although in this case, the analysis of the metric products generated from thermal data did not reveal any particular anomalies or elements of particular diagnostic relevance, it should be underlined that the use of this strategy—which enables the spatial referencing of thermal images—has the potential to represent an opportunity for the detection and classification of degradation. Traditionally, thermal images are already used for this purpose (e.g., for the detection of moisture or other types of damage that involve surface elements). However, the possibility of spatially and three-dimensionally referencing these images greatly contributes to enhancing this last aspect.

**Figure 19:**
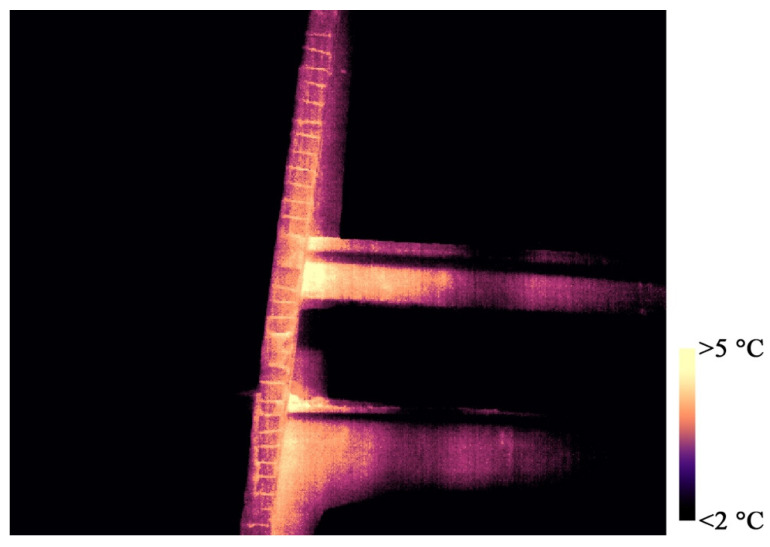
TIR image evidencing a thermal discrepancy between the uncovered irons and the concrete of the arch.

Moreover, the use of this type of imagery can represent a powerful alternative when the purpose is not only to identify but also to classify certain types of degradation. As an example, in Figure 19, it is possible to observe a portion of the intrados of the concrete arch of Morano. By exploiting the difference in terms of emissivity—leading to different temperature values—that characterizes the uncovered irons and the cement surface, it was possible to unambiguously identify, and consequently classify, the portion of the image occupied by the exposed steel bars by segmenting the pixels belonging to this class.

## 6. Conclusions

The present paper highlighted the importance of a structured procedure to integrate the information coming from different techniques. In particular, the combined use of the experimental data was exploited, first, to provide the proper inputs and strategy for the modeling, and second, to assess the health state of the analyzed structure. This approach is necessary to provide the best conservation guidelines.

In fact, for the assessment and conservation of concrete heritage structures, a multidisciplinary approach is required. This includes archival research, 3D surveys derived from image and range-based techniques, and a campaign of non-destructive and destructive testing to understand the behavior and vulnerabilities of these buildings. In detail, a heritage structure requires a process of knowledge divided into stages: general identification of the structure and its environmental factors, collection of geometric and structural data, identification of materials and survey of their state of conservation using historical documentation, mechanical characterization of materials using different investigation techniques, and soil and foundation analyses and related monitoring activities. The documentation process concerns aspects such as construction defects, irregularities, degradation, damage caused by previous events, and any general factor that makes each of these structures unique and involves a greater degree of complexity when interpreting the structural behavior.

It is important to point out that the products of the cognitive process underlying the conservation plans must interpret the results of different investigation techniques in a coordinated way. The possibility of referring the different results to a 3D model, which can constitute a 3D archive of information and, above all, offer the possibility of evaluating the value of the results of the diagnostic tests in their spatial reference, is greatly helpful in keeping under control or facilitating the interpretation of the phenomena.

In this sense, the coordinated experiences of surveying and 3D modeling via innovative techniques and destructive and non-destructive diagnostics tests must seek more and more alliances of intent that are able to raise the degree of understanding and usability of the information derived from the investigations, such as was pursued in this experience of the Morano arch.

The standards also emphasize the importance of periodic building inspections as a primary tool for conservation. Indeed, monitoring is not only a method to investigate the structure’s past but can play an active role in the conservation of historic buildings and influence the decision-making process. This paper, in particular, presents an integrated approach to a reinforced concrete heritage structure, namely, the parabolic arch of Morano sul Po. In detail, the combination of information gathered from 3D mapping and non-destructive testing resulted in the corroboration of a model to be employed for the preservation of this industrial heritage, also in terms of guidelines for its conservation.

## Figures and Tables

**Table 1:** RMSE of the GCPs and CPs after the bundle block adjustment.

	*X [m]*	*Y [m]*	*Z [m]*	*3D [m]*
GCPs (28)	0.006	0.006	0.008	0.012
CPs (6)	0.009	0.004	0.009	0.013

**Table 3:** Parameters for the formulations of the SONREB method.

Method	Coefficient		
	*a*	*b*	*c*
Method 1: RILEM, 1993 [30]	9.27 × 10^−11^	2.6	1.4
Method 2: J. Gasparik, 1992 [31]	8.06 × 10^−8^	1.85	1.246
Method 3: Di Leo and Pascale, 1994 [32]	1.20 × 10^−9^	2.446	1.058

**Table 4:** DJI Zenmuse XT2 principal specifications.

DJI Zenmuse XT2 (FLIR Tau 2) 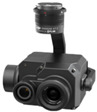	
Type of camera	UAV thermal camera
Thermal imager	Uncooled VoX Microbolometer
Focal length (thermal sensor)	13 mm
Image size (thermal sensor)	640 × 512 pixels
Spectral band	7.5 to 13.5 µm
Temperature range	−25 to 135 °C
Thermal accuracy	±2 °C, ±2%
Thermal sensitivity	0.05 °C at 30 °C
Visible sensor	Embedded
Focal length (visible sensor)	8 mm
Sensor size (visible sensor)	1/1.7″ CMOS
Image size (visible sensor)	4000 × 3000 pixels
Pixel size	1.90 µm

**Table 5:** RMSEs of the GCPs and CPs after the bundle block adjustment: (A) visible dataset processing; (B) thermal dataset processing.

		RMSE			
		X [m]	Y [m]	Z [m]	XYZ [m]
(A)	GCPs (8)	0.004	0.002	0.003	0.005
	CPs (4)	0.004	0.003	0.004	0.007
(B)	GCPs (8)	0.019	0.015	0.013	0.027
	CPs (4)	0.029	0.023	0.023	0.043

**Table 6:** Parameters for the formulations of the SONREB method.

ID Materials	Element	Mechanical Values		
		E [N/m^2^]	Poisson’s Ratio ν [—]	ρ [kg/m^3^]
Beams	Shell	3.04 × 10^+10^	0.2	2500
Top beams	Beams	3.16 × 10^+10^	0.2	2500
Arches	Solid	2.93 × 10^+10^	0.2	2500

## Data Availability

Data are available from the Authors upon request.
